# Compromised quality of life in adult patients who have received a radiation dose towards the basal part of the brain. A case-control study in long-term survivors from cancer in the head and neck region

**DOI:** 10.1186/1748-717X-7-179

**Published:** 2012-10-29

**Authors:** Elisabet Löfdahl, Gertrud Berg, Karl-Axel Johansson, Maria Leonsson Zachrisson, Helge Malmgren, Claes Mercke, Erik Olsson, Lena Wiren, Gudmundur Johannsson

**Affiliations:** 1 Departments of Oncology, Gothenburg, Sweden; 2 Departments Radiation Physics, Gothenburg, Sweden; 3 Department of Endocrinology, Institute of Medicine, Sahlgrenska Academy, University of Gothenburg, Sahlgrenska University Hospital, Grona straket 8, SE-413 45, Gothenburg, Sweden; 4 Department of Philosophy, Linguistics and Theory of Science, Gothenburg, Sweden; 5 Institute of Neuroscience and Physiology, Sahlgrenska University Hospital, Sahlgrenska Academy and University of Gothenburg, Gothenburg, Sweden

**Keywords:** Growth hormone, Head-and-neck cancer, Quality of life, CNS, Low-dose-radiation, Long-term survivors

## Abstract

**Background:**

Adult patients with hypothalamic-pituitary disorders have compromised quality of life (QoL). Whether this is due to their endocrine consequences (hypopituitarism), their underlying hypothalamic-pituitary disorder or both is still under debate. The aim of this trial was to measure quality of life (QoL) in long-term cancer survivors who have received a radiation dose to the basal part of the brain and the pituitary.

**Methods:**

Consecutive patients (n=101) treated for oropharyngeal or epipharyngeal cancer with radiotherapy followed free of cancer for a period of 4 to10 years were identified. Fifteen patients (median age 56 years) with no concomitant illness and no hypopituitarism after careful endocrine evaluation were included in a case-control study with matched healthy controls. Doses to the hypothalamic-pituitary region were calculated. QoL was assessed using the Symptom check list (SCL)-90, Nottingham Health Profile (NHP), and Psychological Well Being (PGWB) questionnaires. Level of physical activity was assessed using the Baecke questionnaire.

**Results:**

The median accumulated dose was 1.9 Gy (1.5–2.2 Gy) to the hypothalamus and 2.4 Gy (1.8–3.3 Gy) to the pituitary gland in patients with oropharyngeal cancer and 6.0–9.3 Gy and 33.5–46.1 Gy, respectively in patients with epipharyngeal cancer (n=2). The patients showed significantly more anxiety and depressiveness, and lower vitality, than their matched controls.

**Conclusion:**

In a group of long time survivors of head and neck cancer who hade received a low radiation dose to the hypothalamic-pituitary region and who had no endocrine consequences of disease or its treatment QoL was compromised as compared with well matched healthy controls.

## Background

Hypopituitarism in adults is mostly due to hypothalamic-pituitary tumours and its treatment with surgery, radiotherapy or both. Although carefully replaced with glucocorticoids, thyroxine and sex steroids, hypopituitarism and untreated growth hormone (GH) deficiency in adults has been associated with compromised quality of life (QoL) [1]. The reduced well-being experienced by many patients with hypopituitarism and untreated GH deficiency has been one of the most compelling arguments supporting GH replacement in adults [2]. This has, however, been questioned by some studies suggesting that the pituitary disease itself or the choice of therapy may explain the reduced QoL in adult patients with hypopituitarism and untreated GH deficiency [3].

Radiation and radiotherapy is an important cause of hypopituitarism and GH deficiency in adults [4]. When brain areas are included in the radiation field, late reactions with vascular and neuronal damage will occur. It is well established that doses of more than 18–20 Gy to the hypothalamic-pituitary area will cause neuroendocrine consequences and reduced QoL and cognitive function. This is particularly well established in adult patients with pituitary adenomas who have received radiotherapy in doses approximating 40 Gy [1]. These patients suffer from low energy levels, lack of vitality, mental fatigue, poor memory and concentration, and increased anxiety and emotional reactions [1]. In such patients it is not possible to differentiate to what extent the effects on QoL and cognitive function are endocrine consequences of radiotherapy, and to what extent they are due to radiation-induced brain damage in the hypothalamus.

The endocrine consequences of radiotherapeutic effects on the hypothalamus are dose related [5]. Although low dose exposure (<100mGy per fraction) has recently been suggested to cause cognitive impairment in children [6], information is unavailable on such low dose exposure in adults. Estimations concerning an increase in secondary malignancies after low dose have been made [7], but other biological late effects, such as endocrine effects or factors known to affect QoL, have not been well studied.

The prognosis for patients with cancers of the head and neck has improved during recent years allowing for a more long-term follow-up of these patients. The causes of this include the introduction of hyper fractionated accelerated radiotherapy (EBRT) and brachytherapy (BT) in combination with chemotherapy [8]. BT is often replaced by intensity modulated radiotherapy (IMRT). Irrespective of the radiotherapy technique used in patients with cancers of the head and neck, a small proportion of the dose will reach the basal part of the brain. Earlier studies have shown disease specific long-term consequences of the disease and its treatment [9-12]. However, in these studies the neuroendocrine consequences of radiation to the basal part of the brain have not been considered as a confounder.

The present patient population was therefore used as a model to explore our research hypothesis that a relatively low dose of radiotherapy towards the adult basal brain will have a long-term impact on QoL independently of the possible neuroendocrine consequences of such therapy. The data from this study of long-term survivors of head and neck cancer may support this hypothesis.

## Methods

### Patients

**Figure 1:**
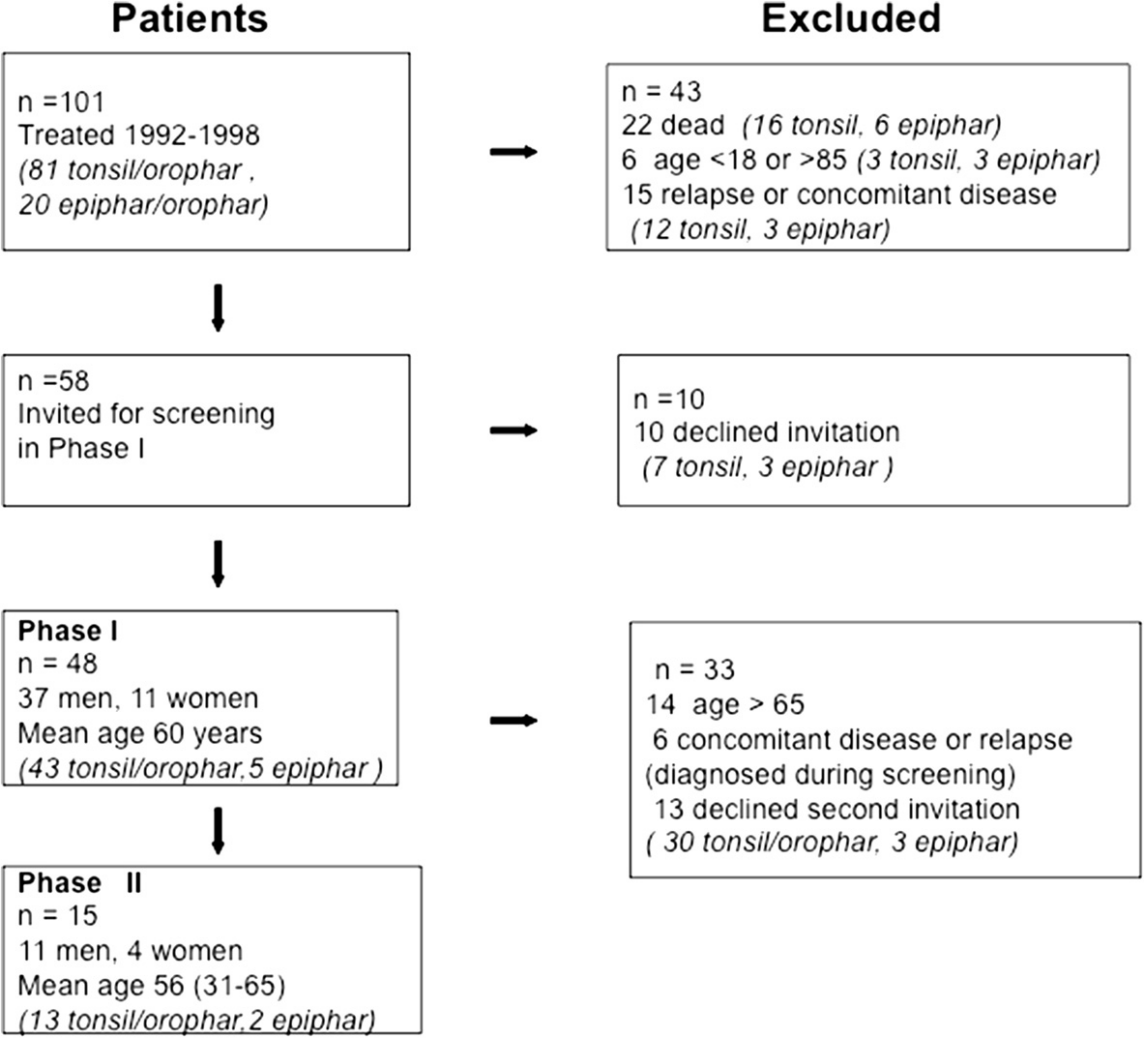
**The trial profile. **From the patient group participating in the screening, survivors were selected in order to make an extended case-control study. In this process patients more than 65 years or with any concomitant disease discovered during the screening process were excluded. Median time from radiation treatment to the performance of the study was 6 years with range 4-10 years. For characteristics of the patients in the case control-study, see Table 1.

From the local database of the Department of Oncology in 2002, 101 individuals with head and neck cancers were identified. Adults between 20 and 85 years of age who had received radiotherapy for their cancer 4–10 years earlier were eligible for the study. All patients had received radiotherapy to the neck and the base of the skull; the histopathology for all tumours was squamous cell carcinoma. Patients were excluded for further analysis if they had significant nutritional difficulties or any other major consequences after previous treatment of the primary disorder, had significant pulmonary disease or active malignancy, or were dependent regarding activities of daily living. Patients with a known neuroendocrine disorder, such as a gross hypothalamic-pituitary lesion or previously verified anterior pituitary hormone deficiency, were also excluded from further analysis (Figure 1).

Using the above selection criteria, 58 survivors were identified and invited to attend a pre-study visit to finalize their inclusion in the study. Clinical endocrine and evaluation was performed. Forty-eight patients came to this first visit. Fifteen patients without endocrine or concomitant disorders or relapse of cancer then consented to be included in the case-controlled study. Median time from radiation treatment to performance of the study was 6 years (range 4–10 years). See Table 1 for a further summary of patients’ characteristics.

**Table 1:** **Patient characteristics, treatment and, radiotherapy doses in growth hormone sufficient adults who have received a radiation dose towards the basal part of the brain**^**1**^

**Pat no**	**Sex and age**	**Diagnose/tumour stage**	**Chemo-therapy**	**Tumour BT Boost (Gy)**	**Tumour/ Neck (r-l) EBRT Dose (Gy)**	Hypo- thalamus EBRT + BT* Dose (Gy)	**Pituitary EBRT + BT Dose (Gy)**
1	m 32	Tonsil /T1N0M0	No	25	40.8 /40.8-0	0.9 + 0.7 =1.6	1.3 +0.7= 2.0
2	m 56	Tonsil/T3N3M0	Yes	12	64.6 /64.6-40.8	1.6 + 0.3 = 1.9	2.2 +0.3 = 2.5
3	f 53	Tonsil/T2N0M0	No	27	40.8 /40.8-40.8	1.1 + 0.7 = 1.8	1.3 +0.8 = 2.1
4	f 51	Epipahr/T2N1M0	Yes	6	61.2 /61.2-40.8	8.1 + 1.2 = 9.3	44.2 +1.9 = 46.1
5	m 50	Tonsil/T1N1M0	Yes	25	40.8 /40.8-64.6	1.2 + 0.7 = 1.9	1.7 +0.7 = 2.4
6	m 66	Epipahr/T2N0M0	No	6	61.2 /40.8-40.8	4.8 + 1.2 = 6.0	31.6 +1.9 = 33.5
7	m 55	Tonsil/T3N0M0	Yes	15	64.6 /40.8-40.8	1.8 + 0.4 = 2.2	2.3 +0.4 = 2.7
8	f 54	Tonsil /T2N2M0	Yes	27	40.8 /40.8-64.6	1.2 + 0.7 = 1.9	1.7 +0.8 = 2.5
9	m 55	Tonsil/T3N0M0	Yes	12	64.6 /40.8-40.8	1.5 + 0.3 = 1.8	2.1 + 0.3 = 2.4
10	m 59	Orophar/T2N0M0	No	0	50.0 /50.0-50.0	2.0 + 0 = 2.0	2.7 + 0 = 2.7
11	m 58	Necklgl/TxN1M0	Yes	0	64.6 /40.8-64.6	2.0 + 0 = 2.0	3.3 + 0 = 3.3
12	m 65	Tonsil/T4N0M0	Yes	12	64.6 /40.8-40.8	1.2 + 0.3 = 1.5	1.9 + 0.3 = 2.2
13	m 66	Tonsil/T2N2M0	Yes	12	64.6 /40.8-64.6	1.5 + 0.3 = 1.8	2.1 + 0.3 = 2.4
14	f 63	Tonsil/T2N0M0	No	26	40.8 /40.8-40.8	0.8 + 0.7 = 1.5	1.0 + 0.8 = 1.8
15	m 61	Tonsil/T4N0M0	Yes	12	64.6 /40.8-40.8	2.0 + 0.3 = 2.3	2.6 + 0.3 = 2.9

^1^Abbreviations: BT = brachytherapy, r = right side of neck, l = left side of neck, EBRT = external beam radiotherapy, m = male, f = female.

* The brachytherapy dose contribution was estimated to be 1.2 Gy to the hypothalamus and 1.9 Gy to the pituitary for patients 4 and 6. For all other patients, brachytherapy doses to the hypothalamus was 2.7% and to the pituitary 2.9%, estimated from calculations performed on 6 patients with cancer in the tonsil.

Fifteen healthy controls matched for age, sex, BMI, and social status were recruited. Relatives or close friends were selected as controls in order to adjust for socioeconomic status. Oral and written informed consent was obtained from patients and controls before entering the study. The study was approved by the Ethics Committee of the University of Gothenburg (No. S644-01).

### Cancer treatment

All patients had received external-beam radiotherapy (EBRT) with a beam quality of 4–6 MV from linear accelerators (Varian) using CT-assisted 3-D dose planning (Cadplan System). The primary target volume was defined as gross target volume (GTV) with a margin of 1.5–2 cm. An elective target volume was also defined, mainly in the neck region. This means that treatment was divided into two parts, one cranial and one lower (neck), with a division at the level of the hyoid bone. In the lower volume there was a central shielding of the larynx, which also meant shielding of the central part of the thyroid gland. In treatment of the oropharynx the cranial border is at the level of the palate, and when treating the epipharynx the border is at the top of the sella turcica. The dose from EBRT was either 40.8 Gy or 64.6 Gy (specified at the isocenter) to the primary target volume according to tumour stage by use of hyper fractionated, accelerated fractionation. The schedule used was 1.7 Gy twice a day. There was a pause for 8–10 days after either 34.0 or 40.8 Gy. The dose to the elective volume was 40.8 Gy, delivered in the same fractionation schedule.

In addition to EBRT, 13 patients who qualified for the case-controlled phase received a brachytherapy (BT) boost after the EBRT. BT was delivered with an interstitial ^192^Ir wire-loop technique, using low dose rate. The target volume was gross target volume (GTV) with a margin of some millimetres. The BT dose was prescribed as 85% of the mean dose calculated in centrally located points and a total dose of 6-26 Gy was given depending on tumour stage.

**Figure 2:**
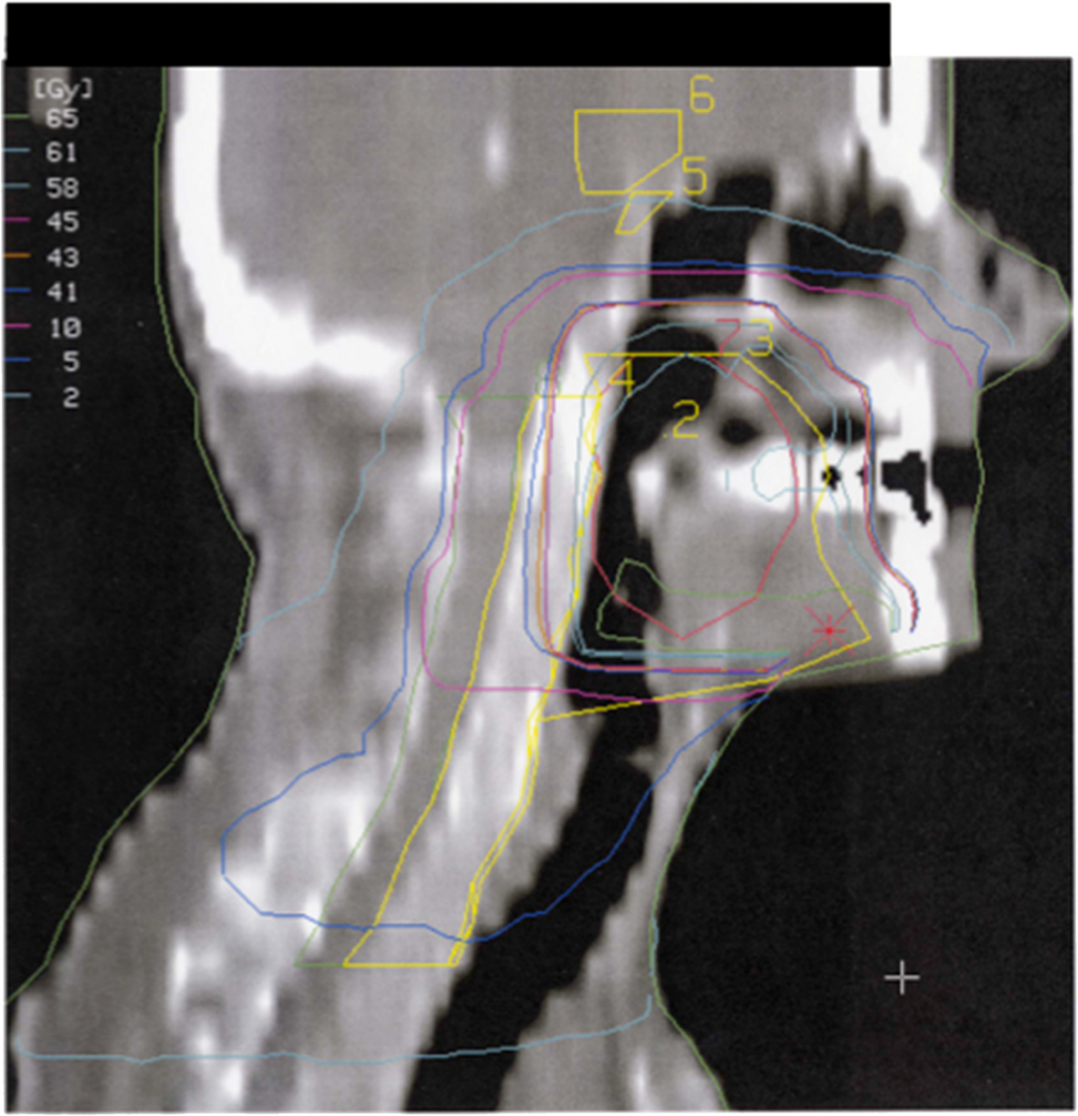
**The sagittal view of the dose distribution of the external beam radiotherapy from patients with cancer of the tonsil. **Volumes of interest and isodose lines are presented. Abbreviations: 2= Gross target volume (GTV), 3=Planning target Volume (PTV), 4=medulla, 5= pituitary, 6= hypothalamus.

**Figure 3:**
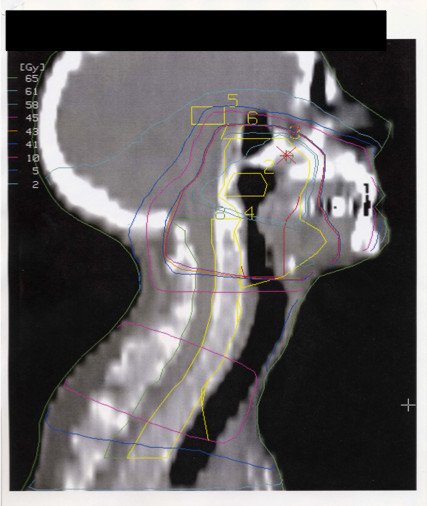
**The sagittal view of the dose distribution of the external beam of patients with cancer of the epipharynx. **Volumes of interest and isodose lines are presented. Abbreviations: 2= Gross target volume (GTV), 3=Planning target volume (PTV) 4=medulla, 5= pituitary, 6= hypothalamus.

For all T3–T4 and N positive tumours, the dose to the primary target aimed at 64 Gy. For T1 and T2 sites, EBRT was stopped at 40.8 Gy followed by BT, which delivered an additional dose of 20–25 Gy toward the target. For T3 and T4 tumours, an external dose of 64.6 Gy was boosted with BT with an additional 10–12 Gy. All node-negative sites had EBRT stopped at 40.8 Gy (Figures 2 and 3). BT techniques were used as previously described [13]. The balance between the dose delivered by EBRT and BT was determined for sparing normal tissue around the tumour (e.g., the salivary glands, oral mucosa and mandible) for T1 and T2 tumours. T3 and T4 tumours are considered to be so bulky that a higher dose than is generally considered reasonable to deliver with EBRT should be given; BT in this region can be considered the ultimate boost [14].

Patients with stage T2N1–T4 tumours (n=35 at screening and n=10 in the case-controlled study) also received one cycle of induction chemotherapy with platinol 100 mg/m^2^ day 1 and 5-fluorouracil (5-FU) 1000 mg/m^2^ day 1–5 three weeks prior to radiotherapy.

### Determination of the dose to the basal part of the brain

**Figure 4:**
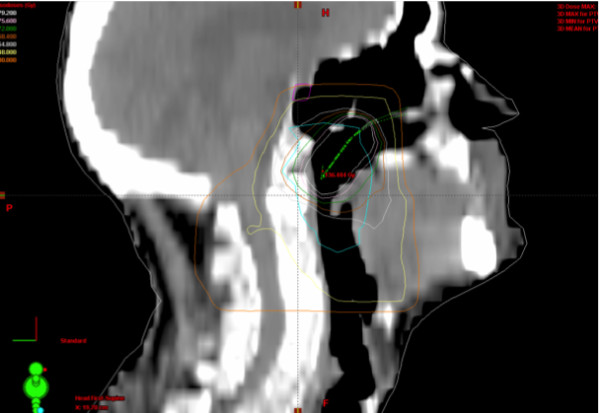
**The sagittal view of the dose distribution of the sum of external beam radiotherapy and the brachytherapy in patients with cancer of the epipharynx. **Volumes of interest and isodose lines are presented. Abbreviations: PTV-T= Planning target volume for the external beam.

In the 15 patients in the case-controlled phase of the study, the total dose (i.e., the dose from both the EBRT and the BT) was calculated from the CT-based dose plans (Figure 4).

The two volumes of interest (hypothalamus and pituitary) were identified from the CT scans. The dose to the basal part of the brain, pituitary, and hypothalamus was calculated. For seven patients, including the two epipharynx patients, the mean EBRT dose to the hypothalamus and pituitary was determined from the treatment planning system. EBRT was individually planned in three dimensions for these patients, based on the CT imaging results. For the other eight patients, the distances from the field edges to the organs of interest were determined from simulator films. Different methods were used because of changes in the planning system. The doses were calculated at several distances from the edges, based on results from the first seven patients and standard tables. The photon beam quality was 6 MV. For BT the mean dose to the hypothalamus and pituitary was reconstructed on 3D CT slice geometry for a few typical patients with epipharynx and tonsil tumours. The mean BT dose to the organs of interest was then added to the dose from the external beams (Figure 4). Tumours of the oropharynx were treated with a low dose rate source, while tumours of the epipharynx were treated with a high dose rate source. The uncertainty at the 95 % confidence level of the total mean doses to the hypothalamus and pituitary has been estimated to be approximately 15%.

### Quality of life (QoL)

The QoL was assessed using four generic self-rating questionnaires: The Symptom Check List-90 (SCL-90) [15], the Nottingham Health Profile (NHP) [16], the Psychological General Well-Being (PGWB) index [17] and the Baecke questionnaire. SCL-90 is a 90-item multidimensional self-report inventory designed to screen for a broad range of psychological problems and symptoms of psychopathology. With the NHP, the patients answer “yes” or “no” to 38 questions concerning problems with emotional reaction, sleep, energy, pain, physical mobility, and social life. The results are used to calculate scores for each of these domains, as well as an overall total score. High scores denote severe problems and a low QoL.

The PGWB contains 22 questions, the answers to which generate both an overall score and six sub-scores covering anxiety, depression, well-being, self-control, health, and vitality. Patients answer each question by selecting a number from 1 to 6, 1 being the most negative option and 6 the most positive. Therefore, in contrast to the NHP, low PGWB scores denote poor QoL. Finally, the Baecke questionnaire was used to assess daily physical activity [18]. Baecke is a self-administered questionnaire (29 items) about habitual activities that gives information on three factors: physical activity at work, sport during leisure time, and physical activity during leisure time excluding sport.

### Biochemistry

To determine the adequacy of the hypothalamic-pituitary axis and thyroid function, the following analyses were performed for the 48 patients participating in the first study visit: serum insulin-like growth factor (IGF)-I, prolactin, testosterone (in men), gonadotropins (LH, FSH), free thyroxine, thyroid stimulating hormone (TSH), and serum cortisol at 0900 hrs. Commercial in-house immune assays were used for all analysis. Patients with abnormal levels in any of these hormones were further evaluated. The 15 patients who entered the case-controlled study were also tested for GH deficiency using an insulin tolerance test.

### Statistics

Values are presented as mean and SD. The statistical significance of the difference between cancer patients and matched controls was calculated using Wilcoxon`s signed rank test, as patients and controls were individually matched. A p-value ≤ 0.05 was considered statistically significant.

## Results

### Endocrine evaluation

All patients in the case-control study had a peak GH response above 6.0 μg/L (abnormal response < 3μg/L). The mean response to the insulin-induced hypoglycaemia among the patients was 13.5 μg/L, ranging between 6 and 42. The patients and controls had also similar serum concentrations of sex steroids and free T4 (12.0 ± 0.6 vs. 12.9 ± 0.4 pmol/L) and TSH (1.40 ± 0.18 vs. 1.38 ± 0.14 mIU/L) although the thyroid hormones were statistically different between the groups (both p<0.05).

Of the 48 patients who took part in the pre-study visit, three already had well controlled T4 replacement therapy due to primary hypothyroidism. An additional 13 patients were diagnosed with primary hypothyroidism during the trial (clinical signs and TSH > 4 mIU/L). Nine of the hypothyroid patients had mild hypothyroidism with TSH levels between 4 and 8 mI U/L. Primary hypothyroidism was considered secondary to neck radiation [19,20]. The 13 newly identified hypothyroid patients received thyroxin replacement therapy. When patients had been clinically and biochemically euthyroid for at least 6 months, they were eligible to enter the case-controlled study. Therefore, three of the newly substituted patients were included in the case-controlled study, and a total of six patients had thyroxin replacement in the study.

We found one patient with severe hypopituitarism who was excluded from further analysis. No other overt pituitary insufficiency was found during the screening procedure. Prolactin was slightly elevated in seven individuals and slightly low in one.

### Dose calculation

The median dose to the hypothalamus was 1.9 Gy (range 1.5–2.2 Gy) and the median dose to the pituitary gland was 2.4 Gy (range 1.8–3.3 Gy) in the 13 patients with oropharyngeal cancer. The pituitary gland is to a large extent included in the irradiation field in the epipharynx patients. The two patients with epipharyngeal cancer therefore received 6.0 and 9.3 Gy to the hypothalamus and 33.5 and 46.1 Gy towards the pituitary.

### Quality of life

The Baecke questionnaire demonstrated a trend for reduced physical activity during leisure time for patients compared to controls (p=0.06) (Table 2).

**Table 2:** **Results from the Baecke questionnaire measuring physical activity in growth hormone sufficient adults who have received a radiation dose towards the basal part of the brain and matched controls**^**2**^

**Domains**	**Patients n = 15**	**Controls n = 15**	**p-value**
Work	2.80 (0.46)	2.76 (0.59)	0.8
Sport	2.43 (0.68)	2.83 (0.62)	0.1
Leisure	2.98 (0.56)	3.48 (0.68)	0.06
Total score	8.22 (1.31)	9.08 (1.39)	0.14

^2^Score given as mean value ± one standard deviation.

Patients also had a significantly higher score in the domains of somatisation, depression, anxiety, and global severity index in the SCL-90 questionnaire, demonstrating compromised QoL in patients compared to controls (Table 3). In the NHP domains of emotional reaction and energy, scores were increased in patients compared to controls, meaning that QoL was reduced in patients (Table 4). Patients as compared with controls had reduced PGWB scores, indicating reduced self-perceived QoL in the domains of anxiety, depression, well being, general health, vitality, and total scores (Table 5).

**Table 3:** **Results from the Symptom Checklist (SLC-90) questionnaire in growth hormone sufficient adults who have received a radiation dose towards the basal part of the brain and matched controls**^**3**^

**Domains**	**Patients n = 15**	**Controls n = 15**	**p-value**
Somatisation	0.647 (0.496)	0.357 (0.366)	0.005 (0.013)
Obsessive-Compulsive	0.670 (0.578)	0.447 (0.253)	0.14
Interpersonal sensitivity	0.453 (0.504)	0.313 (0.295)	0.55
Depression	0.753 (0.666)	0.190 (0.167)	0.01 (0.03)
Anxiety	0.40 (0.417)	0.27 (0.492)	0.05 (0.09)
Hostility	0.42 (0.581)	0.22 (0.308)	0.3
Phobic Anxiety	0.11 (0.168)	0.30 (0.082)	0.08
Paranoid Ideation	0.42 (0.64)	0.16 (0.181)	0.33
Psychoticism	0.19 (0.292)	0.04 (0.063)	0.16
Global severity index	0.502 (0.444)	0.243 (0.123)	0.04 (0.08)
Positive symptom distress index	1.0 (1.73)	3.33 (11.8)	0.6

^3^Score given as mean value ± one standard deviation. P-values in brackets are after the exclusion of two patients with epipharynx cancer.

**Table 4:** **Results from the Nottingham Health Profile (NHP) questionnaire in growth hormone sufficient adults who have received a radiation dose towards the basal part of the brain and matched controls**^**4**^

**Domains**	**Patients n = 15**	**Controls n = 15**	**p-value**
Emotional reaction	7.0 (10.3)	0.0 (0.0)	0.02 (0.03)
Sleep	5.8 (10.0)	7.1 (15.3)	0.9
Energy	14.6 (23.0)	0.0 (0.0)	0.04 (0.07)
Pain	4.2 (12.0)	2.5 (9.8)	0.6
Physical mobility	1.4 (3.6)	0.7 (2.6)	0.4
Social isolation	4.7 (13.0)	0.0 (0.0)	0.2
Total score	6.3 (7.6)	1.7 (3.4)	0.08

^4^Score given as mean value ± one standard deviation. High scores reflect low quality of life. P-values in brackets are after the exclusion of two patients with epipharynx cancer.

**Table 5:** **Results from the Psychological General Well-Being (PGWB) questionnaire in growth hormone sufficient adults who have received a radiation dose towards the basal part of the brain and matched controls**^**5**^

**Domains**	**Patients n = 15**	**Controls n = 15**	**p-value**
Anxiety	24.2 (5.0)	27.8 (1.7)	0.02 (0.03)
Depression	16.6 (1.5)	17.8 (0.6)	0.02 (0.03)
Well-being	16.6 (4.0)	20.0 (2.2)	0.01 (0.02)
Self consciousness	16.0 (1.8)	16.7 (1.3)	0.4
General health	14.5 (2.6)	16.6 (2.0)	0.009 (0.01))
Vitality	17.4 (4.7)	21.5 (1.5)	0.006 (0.007)
Total score	105.3 (17.2)	120.4 (6.3)	0.005 (0.006)

^5^Score given as mean value ± one standard deviation. P-values in brackets are after the exclusion of two patients with epipharynx cancer.

By excluding the patients with the highest doses towards the basal volume of the brain (i.e., the two patients with epipharynx cancer), three out of 12 significant findings in the QoL questionnaires were lost: anxiety (p=0.096) and global severity index (p=0.08) in the SCL-90, and the energy score (p=0.066) in the NHP. Patients receiving thyroxine replacement therapy for primary hypothyroidism (n=6) had similar QOL scoring as patients with normal thyroid function (data not shown).

## Discussion

A novel finding of our study was that in long-term survivors who had received radiation treatment for oropharynx cancer and a low inadvertent accumulated dose towards the basal part of the brain and the pituitary, quality of life was compromised. In order to eliminate several confounders, patients included were highly selected well functioning patients without hypopituitarism and GH deficiency.

The reason for selecting this patient group for long-term studies of the effects of low dose radiation toward the basal part of the brain and the pituitary is that their radiotherapy has been standardised in our unit for the last two decades. Another reason is their favourable long-term prognosis obtained by using intense multimodal treatment that has increased the five-year survival rate, even in patients with advanced T3 and T4 tumours in the head and neck region [14]. In this group of patients it was therefore possible to study the biological late effects after low-dose radiotherapeutic exposure.

The effects of low doses of ionizing radiation in infancy, such as in the treatment of haemangioma, have recently been studied and found to influence cognitive abilities in adulthood when the doses given to the brain were above 100 mGy [6]. Furthermore, adults treated for childhood onset acute lymphatic leukaemia (ALL) with cranial radiotherapy using doses of 18–20 Gy developed GH deficiency and impaired neuropsychological performance, although self-reported QoL was not affected [21]. Adults treated with radiotherapy for cancer of the nasopharynx and paranasal sinuses show radiation-induced vascular damage and cognitive decline at follow-up when total doses >40Gy were administered to the temporal lobe [22]. Long-term studies in adults who have received low-dose radiation towards the brain in adulthood and where the possible neuroendocrine consequences have been accounted for have not been performed previously, to our knowledge. We therefore carefully selected patients with no detected neuroendocrine consequences of previous treatment. We also excluded the presence of GH deficiency using the insulin tolerance test.

We have in our patient group 6 of 15 patients with primary hypothyroidism, a condition that can affect QoL. During inadequate hypothalamic stimulation the pituitary may synthesise and secrete a TSH that is biologically less active and with a longer half-life [23]. This may complicate the diagnosis of hypothyroidism in this patient population as some have received both radiation towards the hypothalamic-pituitary areas and the thyroid gland. However, as TSH synthesis and secretion is more resistant to radiation than GH and gonadotropins [4] the hypothalamic radiation is less likely to be a confounder of importance in this study as all patients tested normal for both GH and gonadotropic function. All patients with primary hypothyroidism were also fully replenished with T4 for more than 6 months before entering the case-controlled trial and their thyroid hormone concentrations and their QoL scoring were similar to those in the healthy matched controls. We therefore believe that the impact of hypothyroidism on the outcome of this study is most likely negligible [24].

In this study, we have determined the total radiotherapeutic doses to the hypothalamus and the pituitary. The hippocampus, which is a radiosensitive structure in the basal part of the brain, is also of interest because it is known that hippocampal damage might affect cognitive function and wellbeing [25]. The hippocampal dose could not be calculated with good accuracy in our study, as the structure cannot be defined well enough on the dose planning CT. We therefore assume that the hippocampus received approximately the same dose as the hypothalamus, as these regions have the same distance from the base of the skull. A volumetric study of the hippocampus has been performed as part of the case-control study in order to further study this possibility [unpublished data].

Many patients treated with head and neck cancer suffer from decreased QoL due to xerostomia, trismus, and swallowing difficulties one to five years after treatment [9,10]. Only a few studies have been performed using generic self-rating QoL questionnaires in patients with no sign of recurrence years after treatment [11,12]. Hammerlid et al. [10] showed that patients still suffered from functional limitations related to the disease and its treatment three years after diagnosis and treatment of head and neck cancer. However, these problems did not generally affect their overall health-related QoL as assessed using disease specific cancer related QoL questionnaires. Others [26-28] have also reported similar findings. Pourel et al. [12] found that physical functioning, role of functioning, and pain score did not differ from the general population. Their findings indicate that coping processes strongly influence QoL in long-term survivors of cancer. Foley et al [29] showed, using semi-structured interviews with survivors of several cancers more than 15 years after their diagnoses that the majority had experienced either a positive influence or very little long-term impact on their lives demonstrating how well cancer patients incorporate the cancer experience into their overall life experience.

Our study addresses general well-being in long-term head and neck cancer survivors (4–10 years) in a case-controlled trial. We have used four generic questionnaires that have all been previously used in various patient groups and in the general population. Although the study is small, the results from three independent questionnaires consistently showed compromised QoL in patients. The various affected domains were also consistent: depression, anxiety, energy, and general well-being. In addition, patients tended to have reduced physical activity during leisure time. Previous studies on QoL in this patient group were cross-sectional case studies, whereas our study was designed as a case-control study also adjusting for socioeconomic status. We have not specifically determined the mechanism for our findings, but a causal relationship between low-dose radiotherapy towards the basal brain and reduced QoL is possible, as the role of several confounders were minimized in the trial design.

## Conclusions

We have demonstrated compromised QoL in patients who had received a low radiation dose towards the basal part of the brain. After eliminating many possible confounders, we suggest that a small amount of absorbed radiotherapeutic dose to the basal parts of the brain has negative long-term consequences in adults that are independent from the neuroendocrine effects of radiation. Our data highlights the importance of further studies on the biological effects of low-dose radiation in normal tissue and volumes at risk [30].

## Competing interests

GJ has received honorarium from NovoNordisk, Pfizer, Eli Lilly, Merck Serono and JCR Pharmaceutical, and consultant fee from AstraZeneca and Viropharma. The authors have no conflict of interest that could be perceived as prejudicing the impartiality of the research reported.

## Authors' contributions

EL, GB, MLZ, LW and GJ contributed to the conception and the design of the trial and performed all the patient related work in the trial. EL, GB, HM and GJ drafted the first version of the manuscript. All authors contributed to the collection of data, data interpretation and critical revision of the manuscript and have reviewed the final version for publication.
